# ABAT and ALDH6A1, regulated by transcription factor HNF4A, suppress tumorigenic capability in clear cell renal cell carcinoma

**DOI:** 10.1186/s12967-020-02268-1

**Published:** 2020-02-24

**Authors:** Jun Lu, Zhan Chen, Hu Zhao, Huiyue Dong, Ling Zhu, Yi Zhang, Jie Wang, Hehuan Zhu, Qiang Cui, Chuang Qi, Shuiliang Wang, Shushang Chen, Jichun Shao

**Affiliations:** 1 Fujian Provincial Key Laboratory of Transplant Biology, Fuzhou General Clinical College, Fujian Medical University, Fuzhou, 350025 China; 2 Fujian Provincial Key Laboratory of Transplant Biology, Dongfang Hospital (900 Hospital of the Joint Logistics Team), Xiamen University, Fuzhou, 350025 China; 3 Department of Urology, 900 Hospital of the Joint Logistics Team, Fuzhou, 350025 Fujian China; 4 Department of Urology, Second Affiliated Hospital of Chengdu Medical College (China National Nuclear Corporation 416 Hospital), Chengdu, 610051 China

**Keywords:** Clear cell renal carcinoma (ccRCC), ABAT, ALDH6A1, HNF4A, Transcription factor

## Abstract

**Background:**

Clear cell renal cell carcinoma (ccRCC) is a malignancy characterized by metabolic reprogramming. ABAT and ALDH6A1 are metabolic enzymes. In this study, we aim to investigate the associations of ABAT and ALDH6A1 with the malignancy of ccRCC cells.

**Methods:**

The gene expression levels of ABAT and ALDH6A1 in ccRCC were analyzed from gene expression microarray datasets and RNA sequencing data. Clinical information was analyzed from The Cancer Genome Atlas (TCGA) data. The distributions of ABAT and ALDH6A1 in ccRCC clinical tissues were screened by reverse transcription-quantitative polymerase chain reaction (RT-QPCR) and immunohistochemical assays. The effect of overexpression of ABAT or ALDH6A1 was measured by detecting the cell viability, migration ability, and the ratio of lactate and nicotinamide adenine dinucleotide phosphate (NADPH). Chromatin immunoprecipitation (ChIP) and luciferase reporter assays were carried out to investigate the transcript regulation of HNF4A in ABAT and ALDH6A1.

**Results:**

Remarkable downregulated ABAT and ALDH6A1 expression levels were observed in ccRCC patients and low expression of ABAT and ALDH6A1 was correlated with poor survival. Overexpression of ABAT or ALDH6A1 significantly attenuated cell proliferation and migration, and impaired lactate production. In ABAT increased ccRCC cells, the ratio of NADPH/NADP+ was reduced. Finally, we demonstrated that ABAT and ALDH6A1 were directly regulated by a tumor suppressor, HNF4A.

**Conclusions:**

These observations identified HNF4A-regulated low-expressed ABAT and ALDH6A1 as promising diagnostic and prognostic biomarkers for ccRCC.

## Introduction

Renal cell carcinoma (RCC) is a malignant tumor that originates from the renal parenchyma urinary tubule epithelia. Clear cell renal cell carcinoma (ccRCC) is the most common pathological type of RCC (60–85%) [1]. Surgical resection is still the first choice for treatment of localized renal cancer [2]. Because kidney cancer is not sensitive to radiotherapy and chemotherapy, some patients who cannot undergo surgical treatment need to consider targeted therapy. Since the US FDA approved sorafenib treatment of metastatic renal cell carcinoma in 2006, it has successively approved a series of drugs, mainly divided into three categories: 1 Tyrosine kinase inhibitors (TKI) categories: sunitinib, pazopanib, etc.; 2 anti-vascular endothelial growth factors: ranibizumab, bevacizumab, etc.; 3 mammalian target of rapamycin (mTOR) inhibitor: everolimus, temsirolimus, etc. [3]. Although targeted drugs prolong the patient’s survival time, their high cost and prolonged use, which leads to the gradual emergence of problems such as drug resistance and toxicity and side effects, limit their widespread use in clinical practice. Therefore, the detailed investigation of the pathogenesis of renal cancer and the search for new targets for targeted therapy of renal cancer are the focus of current research on renal cancer.

Metabolic disorders are one of the important hallmarks of tumors [4]. The proliferation of tumor cells usually requires the re-adjustment of their metabolism to produce enough energy and synthetic raw materials [5]. ccRCC is a tumor characterized by abnormal glucose and lipid metabolism [6]. Numerous studies have shown that multiple pathways associated with gluconeogenesis (e.g., pyruvate metabolism, valeric acid metabolism, arginine and proline metabolism, and degradation of valine, leucine, and isoleucine) are down-regulated in ccRCC [6–8]. ABAT (gamma-aminobutyric acid transaminase) [9] and ALDH6A1 (aldehyde dehydrogenase 6 family, member A1) [10] are involved in the pyruvate metabolism and catabolism of valine, leucine, and isoleucine. Both of them are differential expressed genes (DEGs) of The Cancer Genome Atlas (TCGA)-kidney renal clear cell carcinoma (KIRC) data [11–13]. The role of these two genes in ccRCC development and their gene regulation mechanisms have not been discussed in depth.

Therefore, this study will explore the expression of ABAT and ALDH6A1, their clinical significance and biological functions, correlations between ABAT and ALDH6A1 gene expression, and the transcription factor involved in the regulation of the expression of these two genes in ccRCC.

## Methods

### Clinical specimens

50 cases of renal clear cell carcinoma and matched paracancerous tissues (> 2 cm from the edge of the cancer tissue) were selected from fresh resections of kidney cancer patients at Fuzhou General Hospital from November 2013 to November 2015. Patients were aged 28–77 years, with an average age of 55.5 years. All the cancer tissue specimens were pathologically confirmed as ccRCC. The separation and use of human tissues were approved by the Human Research Ethics Review Committee of Fuzhou General Hospital (Approval No. 2013-017). All patients provided written informed consent. The pathological grades were verified according to the Furhman classification. Additional file 1: Table SI details the patients’ details.

### Cell lines and main reagents

HEK293T cells were purchased from the Shanghai Cell Bank of the Chinese Academy of Sciences (Shanghai, China). ACHN and 786-O cells were provided by Shanghai GK Genentech Co., Ltd. (Shanghai, China). Cells freshly amplified and frozen were used every 2–3 months. They were routinely tested for the absence of mycoplasma contamination. Dulbecco’s modified Eagles medium (DMEM) high glucose medium and RPMI 1640 medium were purchased from Hyclone (Thermo Fisher Scientific, Inc.). Fetal bovine serum was purchased from Gibco (Thermo Fisher Scientific, Inc.). Transfection reagent Lipofiter™ was purchased from HanBio (Shanghai, China). TRIzol was purchased from Invitrogen (Thermo Fisher Scientific, Inc.). A complementary DNA (cDNA) kit was purchased from Fermentas (Thermo Fisher Scientific, Inc.), and a SYBR Green real-time polymerase chain reaction (PCR) kit was purchased from Applied Biosystems (Thermo Fisher Scientific, Inc.). ABAT and ALDH6A1 antibodies were purchased from Sigma-Aldrich (Merck KGaA, Darmstadt, Germany). ACTIN antibody and Flag-tag antibody were purchased from Cell Signaling Technology (Danvers, MA, USA). A water-soluble tetrazolium salt (WST)-1 cell proliferation assay kit was purchased from Roche (Mannheim, Germany). Horseradish peroxidase (HRP)-tagged secondary antibody and ECL kits were purchased from Pierce (Thermo Fisher Scientific, Inc.).

### Gene expression omnibus (GEO) and TCGA data analysis

We selected four ccRCC data sets on Oncomine (http://www.oncomine.org): Beroukhim Renal_GSE14994 [14], Grumz Renal_GSE6344 [15], Jones Renal_GSE 15641 [16], and Lenburg Renal_GSE781 [17]. Down-regulated genes were selected from these four datasets. The data of these four primary dataset were downloaded from the NCBI GEO database (http://ncbi.nlm.nig.gov/geo) [18]. KIRC clinical data and mRNA expression were downloaded from the TCGA website (http://cancergenome.nig.gov) [19]. The transcriptome analysis data file was standardized and then transferred to a txt file using a Perl script. The expression of the ABAT and ALDH6A1 genes is reflected by the intensity of the probe signal. GraphPad Prism 6 software (GraphPad Software, Inc., La Jolla, CA, USA) was used to make gene expression maps. The Kaplan–Meier survival curve of TCGA-KIRC patients was generated on the OncoLnc (http://www.oncolnc.org/) website. A Kyoto Encyclopedia of Genes and Genomes (KEGG, http://www.genome.jp/kegg/) pathway analysis of the DEGs was generated by Metascape (http://metascape.org/gp/index.html#/main/step1) [20]. Gene set enrichment analysis (GSEA) was analyzed in LinkedOmics (http://www.linkedomics.org/login.php#) [21]. Gene expression correlation analysis was obtained from UALCAN (http://ualcan.path.uab.edu/index.html\) [22].

### Quantitative polymerase chain reaction

Total RNA was extracted from tissues using the Total RNA Kit II kit (Omega Bio-Tek, Inc., Norcross, GA, USA). After RNA quantification, 3 μg of total RNA was reverse transcribed into cDNA using the RevertAid First Strand cDNA Synthesis kit. SYBR Green select master mix was used for quantitative polymerase chain reaction (qPCR). The cDNA was subjected to qPCR using ATAT, ALDH6A1, and β-actin primers: ABAT forward primer, 5′-CTTCCGTCTTCATCAGAGGC-3′ and reverse primer, 5′-CAGCTTCCAGCACAGCTACC-3′; ALDH6A1 forward primer, 5′-TGGGACTGGATTTCACCTTG-3′ and reverse primer 5′-GTGCTTCTGGGCAGTAGAGG-3′; and Β-actin forward primer 5′-TGACGTGGACATCCGCAAAG-3′ and reverse primer 5′-CTGGAAGGTGGACAGCGAGG-3′. PCR was conducted for 40 cycles under the following conditions: 94 °C for 30 s, 58 °C for 60 s. Relative fold changes in mRNA expression were calculated by normalization to β-actin mRNA using the formula 2^−∆∆Ct^ [23].

### Western blotting detection

SDS-PAGE electrophoresis was performed at 50 μg/well for each group of proteins quantified by BCA. After electrophoresis, the cells were transferred to PVDF membranes at a constant pressure of 60 V for 180 min. The blocking solution was blocked for 1 h and reacted with the primary antibody at 4 °C overnight; TBST washed three times; HRP-labeled goat anti-rabbit antibody reaction at room temperature for 1 h, TBST washed four times; added ECL luminescence, exposed on an X-ray film, film development, fixing, washing with water, drying, preservation, and photographing.

### Immunohistochemical staining

A tissue microarray (HKid-CRCC060PG-01; Shanghai Outdo Biotech Co., China) was constructed with formalin-fixed paraffin-embedded 29 paired ccRCC and adjacent non-tumor renal tissues. The demographic information was summarized in Additional file 1: Table S2. The paraffin-embedded sections were stained with antibodies against ABAT or ALDH6A1 antibody (dilution at 1:750 or dilution at 1:3000; HPA041528; HPA029073) at 4 °C overnight and incubated with secondary antibody. Negative controls were stained with isotype control IgG. Then, the sections were counterstained with hematoxylin and eosin. Immunohistochemical staining intensity was based on the proportion of cell staining and scored from 0 to 3 following criteria described previously [24]. The slides were analyzed by standard light microscopy.

### Lentivirus transfection

The lentivirus pLVX-Puro-ABAT, pLVX-Puro-ALDH6A1, and control virus pLVX-Puro were purchased from the Public Protein/Plasmid Library (PPL, Nanjing, China). For virus infection, the virus was diluted with serum-containing cell culture medium containing 8 μg/mL polybrene. The fresh medium was changed after 12 h. Puromycin (1 μg/mL, Sigma) was then added to select stable cells (10 days).

### Water-soluble tetrazolium detects cell proliferation

Cells of the observation group, the control group, and the blank group were taken, and the number of cells was adjusted to 2 × 10^3^ cells/ml, and 100 μL cells were inoculated into a 96-well cell culture plate. After 24 h, the cells were adhered and 10 μL of water-soluble tetrazolium (WST-1) reagent was added for 24, 48 and 72 h testing, and incubated for 45 min. Then, the optical density (D450) of each well at 450 nm wavelength was measured with a spectrophotometer (Multiskan GO, Thermo Scientific). Each group set up four complex holes.

### Cell death detection

ABAT, ALDH6A1 stable overexpressed or control ACHN and 786-O cells (1 × 10^5^/well) were plated and grown in 100-mm culture dishes overnight. Cells were harvested and cell death rates were determined using a Cell Death Detection ELISA Kit (Cat. No. 11544675001, Roche, Switzerland) following the manufacturer’s instructions.

### Measurement of lactic acid

Levels of lactic acid were measured using a Lactate Assay Kit (Sigma-Aldrich, USA) following the manufacturer’s instructions. In brief, ABAT, ALDH6A1 stable overexpressed or control ACHN and 786-O cells were treated with Lactate Assay Buffer and the Reaction Mix was added. The reaction mixture was incubated at room temperature for 30 min. The samples were analysed at an absorbance at 450 nm using a spectrophotometer.

### Measurement of NADPH and NADP

Levels of NADPH and NADP+ were measured using a NADP/NADPH Quantitation Kit (Sigma-Aldrich, USA) following the manufacturer’s instructions. In brief, ABAT, ALDH6A1 stable overexpressed or control ACHN and 786-O cells were treated with NADP/NADPH extraction buffer and the NADPH developer was added. The reaction mixture was incubated at room temperature for 1 h. The samples were analysed at an absorbance at 450 nm using a spectrophotometer (Multiskan GO, Thermo Scientific).

### Plasmid and transfection

The pHNF4A-flag and its control plasmid were purchased from the Public Protein/Plasmid Library (PPL, Nanjing, China). HEK293T and ACHN cells were transfected with pHNF4A-flag or control plasmid. At 48 h after transfection, the cells were extracted for quantitative PCR and Western blot assay.

### Luciferase assay

HEK293 cells were plated in 24-well plates and were co-transfected with 100 ng of reporter construct (pGL3-Basic, pGL3-ABAT-promoter, or pGL3-ALDH6A1-promoter), 500 ng of HNF4A expression vector or control vector, and 20 ng of the internal Renilla vector using Lipofiter. After 48 h, the luciferase activity was measured using a dual luciferase reporter assay system (Promega, Madison, WI, USA).

### Chromatin immunoprecipitation assay

HEK293T cells were transfected with pcDNA3.1-HNF4A-flag plasmid using Lipofiter. The chromatin immunoprecipitation (ChIP) assay was performed using a SimpleChIP Plus Enzymatic Chromatin IP Kit (Magnetic Beads) (cell signaling technology) following the manufacturer’s protocol. Cross-linked chromatin was immunoprecipitated with anti-flag rabbit polyclonal antibody (#14793; Cell Signaling Technology) or normal rabbit IgG (Cell Signaling Technology) antibodies. Precipitated DNA was analyzed by quantitative real-time PCR and semi-quantitative PCR. The primers specific to the HNF4A binding region within ABAT promoter region were as follows: forward-1 5′-GTTCATAGGGATTGGATTGCT-3′ and reverse-1 5′-CCCCTTTACTACCCTTGACCT-3′; and forward-2 5′- ATCAACCTATGGAGCTGCCCC -3′ and reverse-2 5′- CTCGCTTTCGCCACAGCCAAT -3′. The primers within ALDH6A1 promoter region were as follows: forward-1 5′-ATCTAGCGCAAAAGGGGCAA-3′ and reverse-1 5′-GCTTTGGGAGACCAAAAGATGAC-3′; and forward-2 5′-ACATCTCCTCCTCGACCCTC-3′ and reverse-2 5′-TCAGCTCACCGGTTCTTCAC-3′.

### Statistical processing

SPSS13.0 software was used for statistical analysis. The data obtained were expressed in terms of mean ± SD. Single-factor repeated-measures analysis of variance found a statistically significant difference at *p* < 0.05.

## Results

### Identification of ABAT and ALDH6A1 as downregulated genes in ccRCC by GEO data analysis

**Fig. 1:**
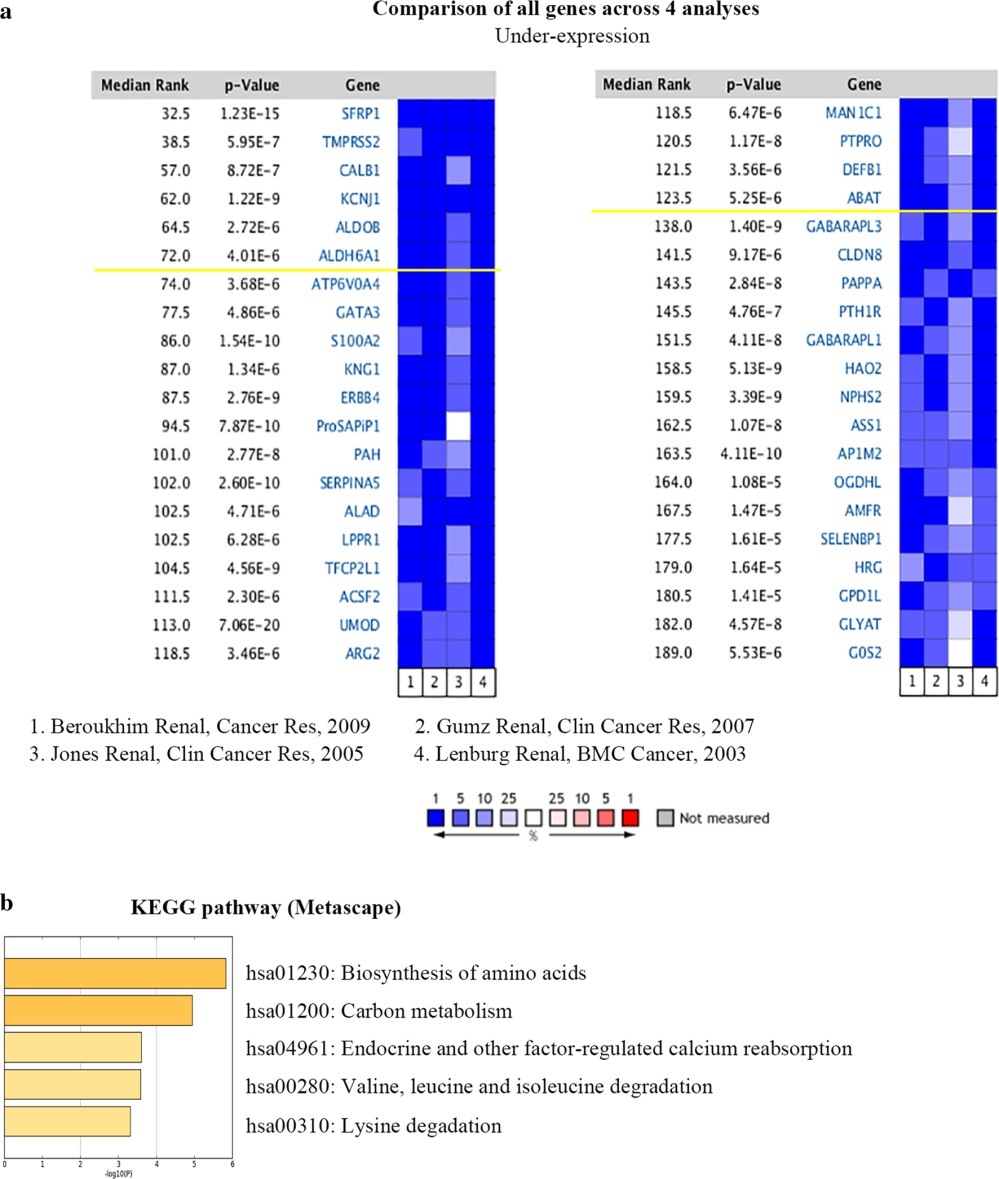
ABAT and ALDH6A1 expressions are downregulated in ccRCC based on Oncomine data mining. **a** Differential expression analysis was performed using Oncomine online analysis tools. **b** KEGG pathway analysis of low expressed DEGs in Metascape. Statistical significance was set at the *p* < 0.05 level. KEGG: Kyoto Encyclopedia of Genes and Genomes

Data mining was performed on the Oncomine database, and four data sets were selected, all of which were detected using the Affymetrix genomic array (Human Genome U133A Array). ABAT and ALDH6A1 were determined to be the downregulated DEGs (Fig. 1a). KEGG pathway enrichment (Metascape) revealed that the top 120 down-regulated DEGs were correlated with the biosynthesis of amino acids, carbon metabolism, endocrine and other factor-regulated calcium reabsorption, valine, leucine and isoleucine degradation, and lysine degradation pathways (Fig. 1b).

**Fig. 2:**
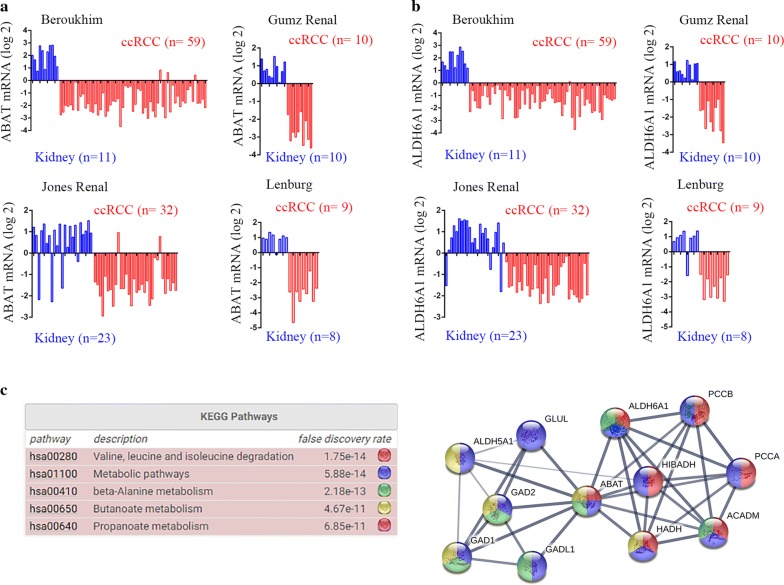
ABAT and ALDH6A1 expression reduced in data analysis of four GEO datasets and assay of their interaction networks. **a** Bar charts of the ABAT mRNA levels (log2) in the four GEO datasets. Blue represents non-tumor tissues and red represents ccRCC tissues. ccRCC, clear cell renal cell carcinoma. **b** Bar charts of the ALDH6A1 mRNA levels (log2) in the four GEO datasets. **c** ABAT and ALDH6A1 interaction genes predicted by STRING analysis

The expression of ABAT and ALDH6A1 was significantly reduced in human ccRCC samples among all four datasets (Fig. 2a, b) (Beroukhim Renal non-hereditary and hereditary ccRCC, n = 59, fold change = − 11.183, *p* < 0.001 (ABAT), fold change = − 9.122, *p* < 0.001 (ALDH6A1); Grumz Renal ccRCC, *n *= 10, fold change = − 10.795, *p* < 0.001 (ABAT), fold change = − 5.828, *p* < 0.001 (ALDH6A1); Jones Renal ccRCC, *n *= 32, fold change = − 3.972, *p* < 0.001 (ABAT), fold change = − 4.413, *p* < 0.001 (ALDH6A1); and Lenburg Renal ccRCC, *n *= 12, fold change = − 11.243, *p* < 0.001) (ABAT), fold change = − 8.680, *p* < 0.001 (ALDH6A1)). Protein association network analysis was performed by STRING. The result shows that ABAT and ALDH6A1 were both involved in the valine, leucine, and isoleucine degradation and beta-alanine metabolism pathways (Fig. 2c).

### ABAT and ALDH6A1 are downregulated in TCGA renal clear cell carcinoma data

**Fig. 3:**
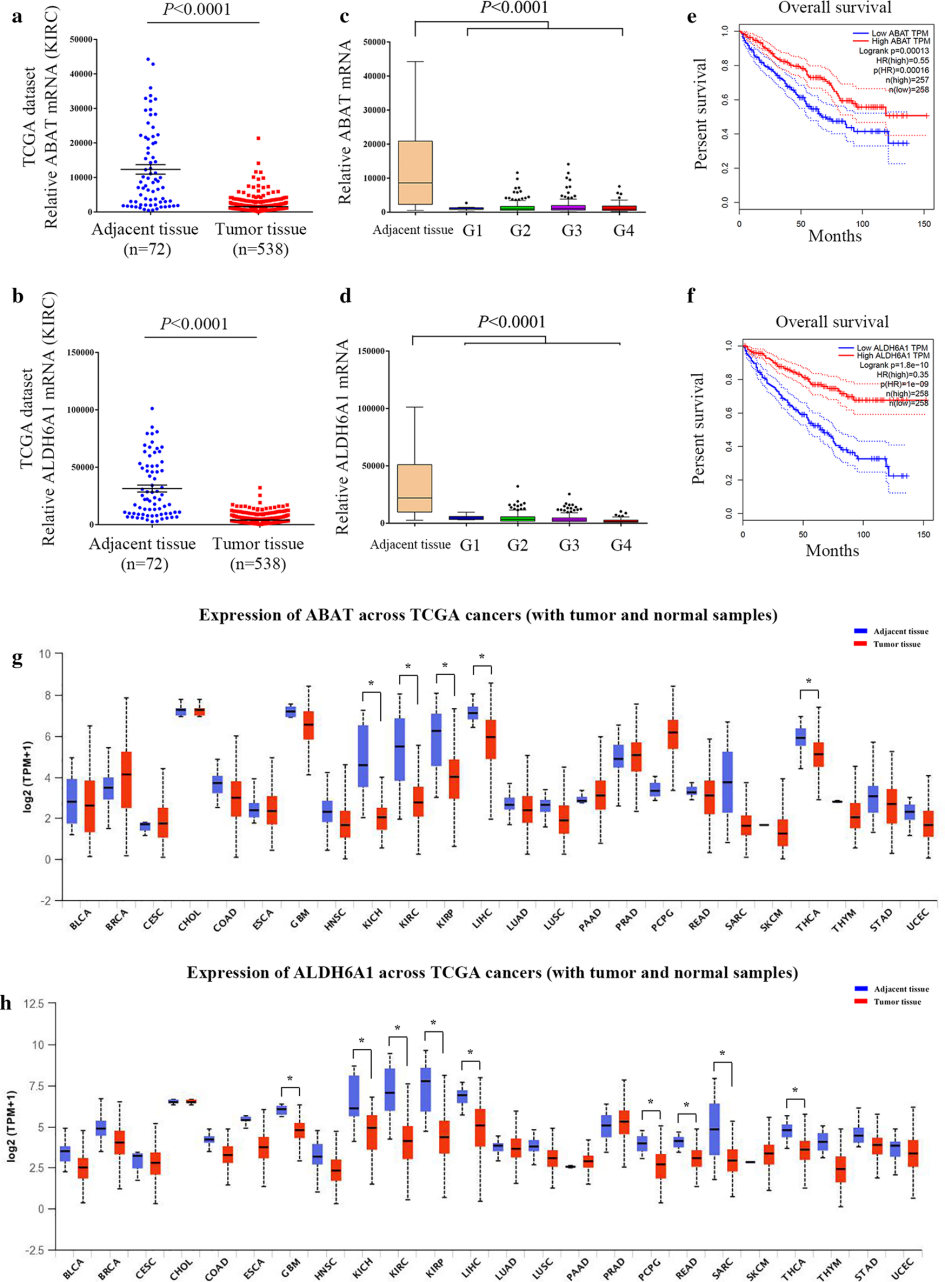
Analysis of renal clear cell carcinoma data in TCGA. **a** Relative expression of ABAT in ccRCC tissues and non-tumor tissues; *p* < 0.0001. **b** Box plots of ABAT mRNA levels in neoplastic tissues, Fuhrman grades Gl, G2, G3, and G4. **c** Survival curves of patients with relatively low and high expression of ABAT in ccRCC patients. **d** Relative expression of ALDH6A1 in ccRCC tissues and non-tumor tissues; *p *< 0.0001. **e** Box plots of ALDH6A1 mRNA levels in neoplastic tissues. **f** Survival curves of ccRCC patients with relatively low and high expression of ALDH6A1. **g** ABAT gene expression profile across all TCGA tumor samples and paired normal tissues. **h** ALDH6A1 gene expression profile across all TCGA tumor samples and their normal tissues. KIRC, Kidney renal clear cell carcinoma

In the TCGA-KIRC dataset, there were 72 paraneoplastic tissues and 538 tumor tissues. The results still showed decreased expression of ABAT and ALDH6A1 in tumor compared with paraneoplastic tissues (*p* < 0.0001) (Fig. 3a, b). The expression of ABAT and ALDH6A1 was decreased in the four pathologically graded tissues, but there was no significant difference between the individual grades, see Fig. 3c, d. Analysis of patient survival revealed that patients with relatively low expression of ABAT or ALDH6A1 tended to have shorter overall survival (Fig. 3e–f). These results suggest that the down-expression of ABAT and ALDH6A1 could be used as biomarkers for ccRCC and that their expression correlates with the survival of patients.

Based on the pan-cancer analysis, both ABAT and ALDH6A1 were significantly down-expressed in three types of renal cell carcinomas, KIRC, kidney renal papillary cell carcinoma (KIRP), and kidney chromophobe (KICH) (Fig. 3g–h). ABAT and ALDH6A1 have similar expression profiles in cancers.

### Expression of ABAT and ALDH6A1 is downregulated in ccRCC clinical samples

**Fig. 4:**
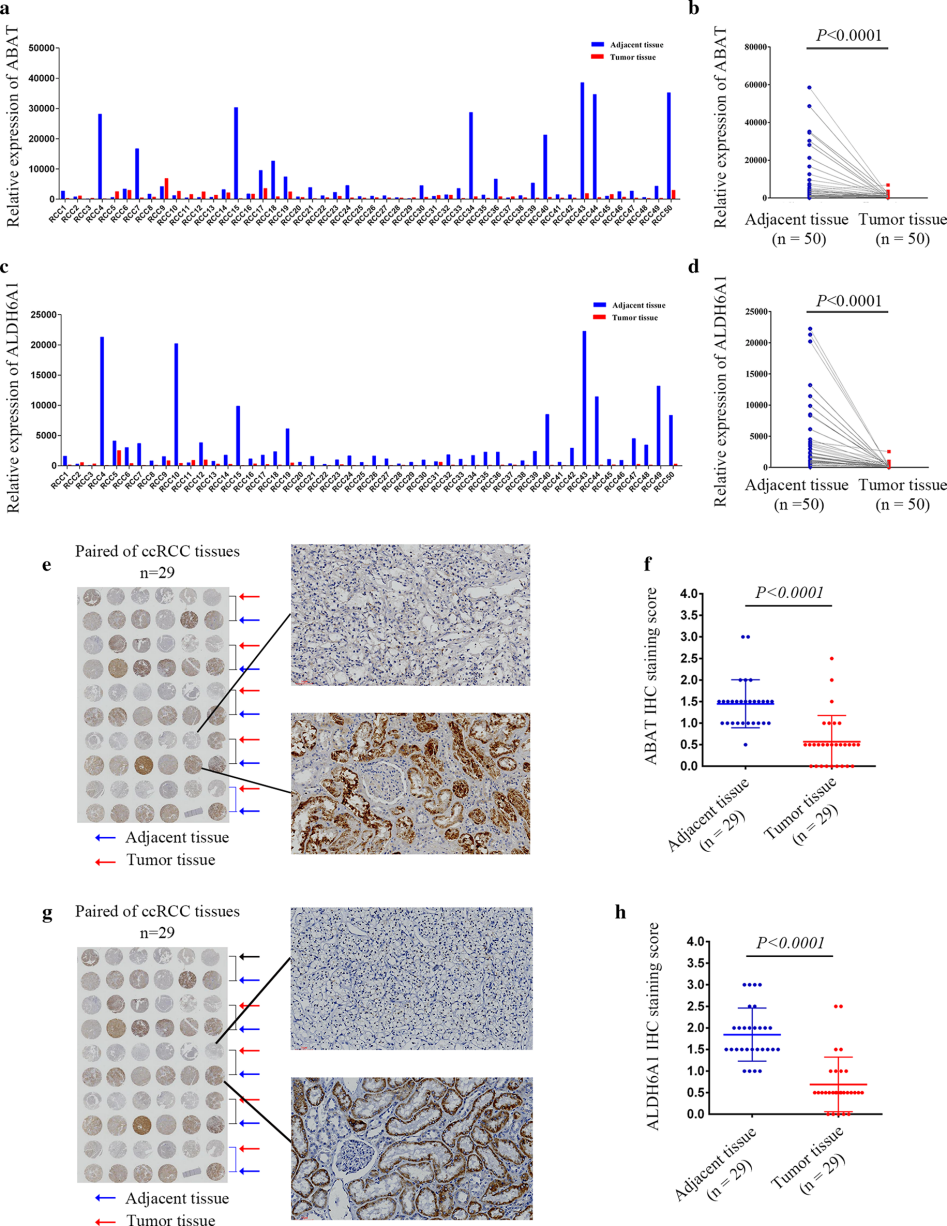
ABAT and ALDH6A1 expression in ccRCC clinical samples. **a** 50 pairs of ccRCC organizations were used to detect ABAT mRNA levels by quantitative-PCR. **b** Paired analysis of ABAT mRNA detection; *p* < 0.0001. **c** For 50 pairs of ccRCC samples, quantitative PCR was used to detect ALDH6A1 mRNA levels. **d** Paired analysis of ALDH6A1 mRNA detection; *p* < 0.0001. **e** Immunohistochemical detection of ABAT. **f** Box plot depicting ABAT levels as assessed by IHC in 29 paired ccRCC samples; *p* < 0.0001. **g** Immunohistochemical detection of ALDH6A1. **h** Box plot depicting ALDH6A1 levels as assessed by IHC in 29 paired ccRCC samples; *p* < 0.0001

From 2013 to 2015, our center collected a total of 50 pairs of pathologically identified and surgically removed ccRCC tumor tissues and their adjacent tissues. Fluorescent quantitative PCR assays showed that ABAT expression was reduced in 94% of tumor tissues (Fig. 4a). The paired analysis showed that ABAT mRNA levels were significantly decreased in renal clear cell carcinoma tissue (*p* < 0.0001) (Fig. 4b). Similar results were observed in ALDH6A1 expression (Fig. 4c, d).

A ccRCC paraffin tissue microarray was used for immunohistochemical detection. Results showed that ABAT protein levels were reduced in ccRCC tissues (Fig. 4e), which reduction was statistically significant (*p* < 0.0001) (Fig. 4f). Similar results were observed for ALDH6A1 protein levels (Fig. 4g–h). These data indicate significant decreases in ABAT and ALDH6A1 expression in the ccRCC clinical samples.

### Overexpression ABAT or ALDH6A1 suppresses renal clear cell carcinoma cell line growth and migration

**Fig. 5:**
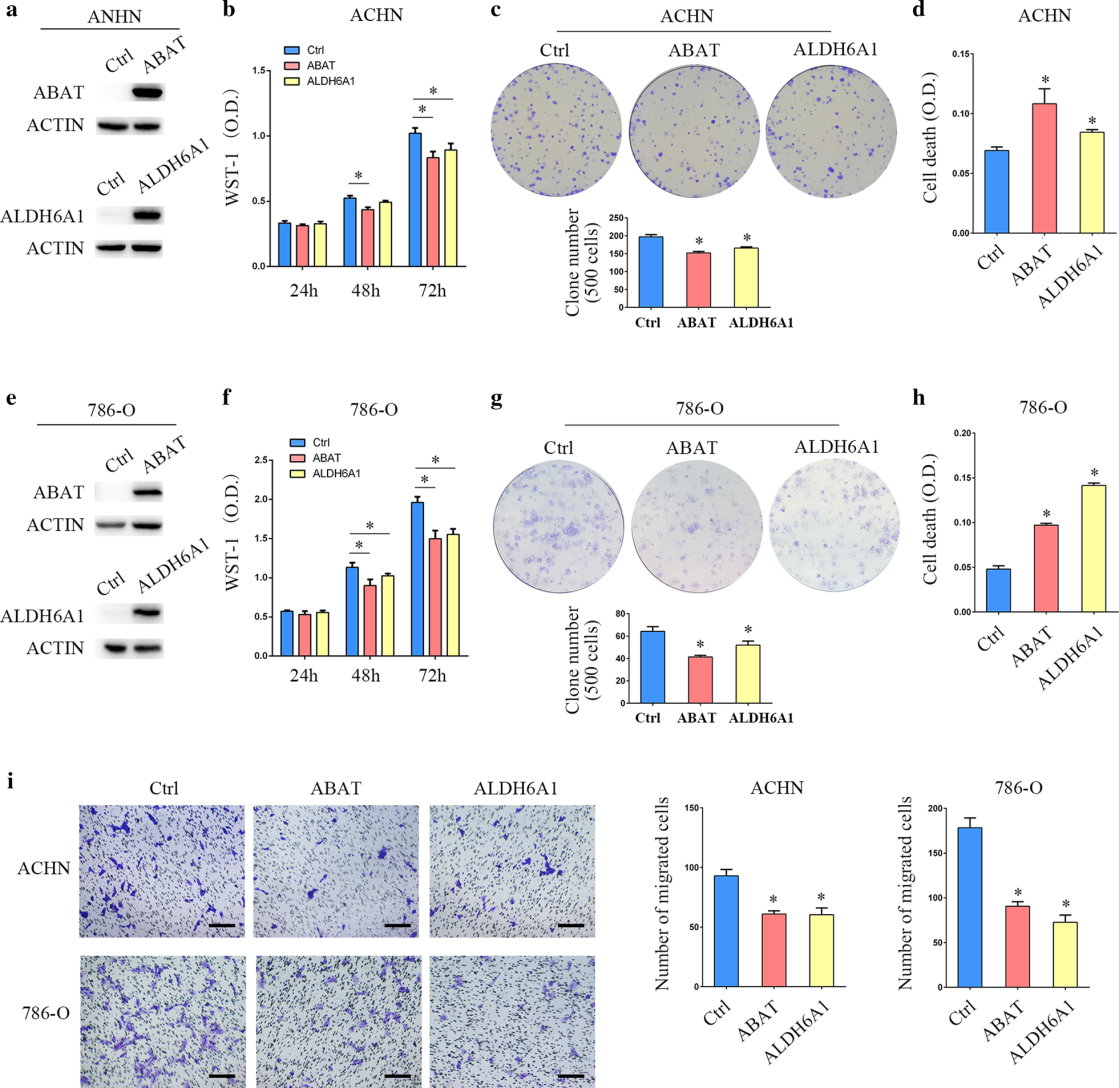
Effects of overexpression of ABAT and ALDH6A1 on ccRCC cell growth. **a** ACHN cells stably transfected with ABAT or ALDH6A1 lentivirus and performed by Western blot analysis. **b** The growth inhibition rates were measured by WST-1 assay kit at 24, 48, and 72 h in ACHN cells that stably expressed ABAT or ALDH6A1. **c** Representative images of clonogenic assays of ACHN cells that stably expressed ABAT or ALDH6A1. **d** ACHN cells stably expressed ABAT or ALDH6A1. Cell death was detected using an ELISA-based cell death detection kit. **e** Western blot assay was performed in ABAT or ALDH6A1 overexpressed 786-O cells. **f** WST-1 assay in 786-O cells that stably expressed ABAT or ALDH6A1. **g** Colony formation assay in 786-O cells that stably expressed ABAT or ALDH6A1. **h** Cell death assay in 786-O cells that stably expressed ABAT or ALDH6A1. **i** Representative images of migration assays of ACHN cells stably expressing ABAT or ALDH6A1 (left) and quantification of the relative migration cell number (right). Scale bar, 100 µm. All experiments were repeated three times. The *p* value was measured using Student’s *t*-test; *p < 0.05, compared with the control cells. Ctrl: cells transfected with empty control lentivirus

We next sought to examine the functions of ABAT and ALDH6A1 in ccRCC cells using a lentivirus-mediated overexpression system. The high expression of ABAT and ALDH6A1 was confirmed in ACHN cells (Fig. 5a). The results of WST-1 assay showed that the proliferation of ABAT and ALDH6A1 overexpression group was significantly lower than that in the control group at 72 h (*p* < 0.05, Fig. 5b). Compared with the control group, the ACHN cells with stable ABAT or ALDH6A1 expression had a significantly reduced ability to form colonies (Fig. 5c). We further assessed the effects of ABAT and ALDH6A1 on cell death. Using cell death ELISA assay, it was observed that overexpression of ABAT and ALDH6A1 increased death rates in ACHN cells (Fig. 5d). Those data were verified in the 786-O cell line, another ccRCC cell line (Fig. 5e–h). Moreover, cell migration was significantly decreased in the ABAT or ALDH6A1 overexpression ACHN and 786-O cells (Fig. 5i). These data collectively indicate that ABAT and ALDH6A1 act as tumor suppressors of ccRCC.

### ABAT and ALDH6A1 overexpression impairs metabolic modeling in ccRCC cells

**Fig. 6:**
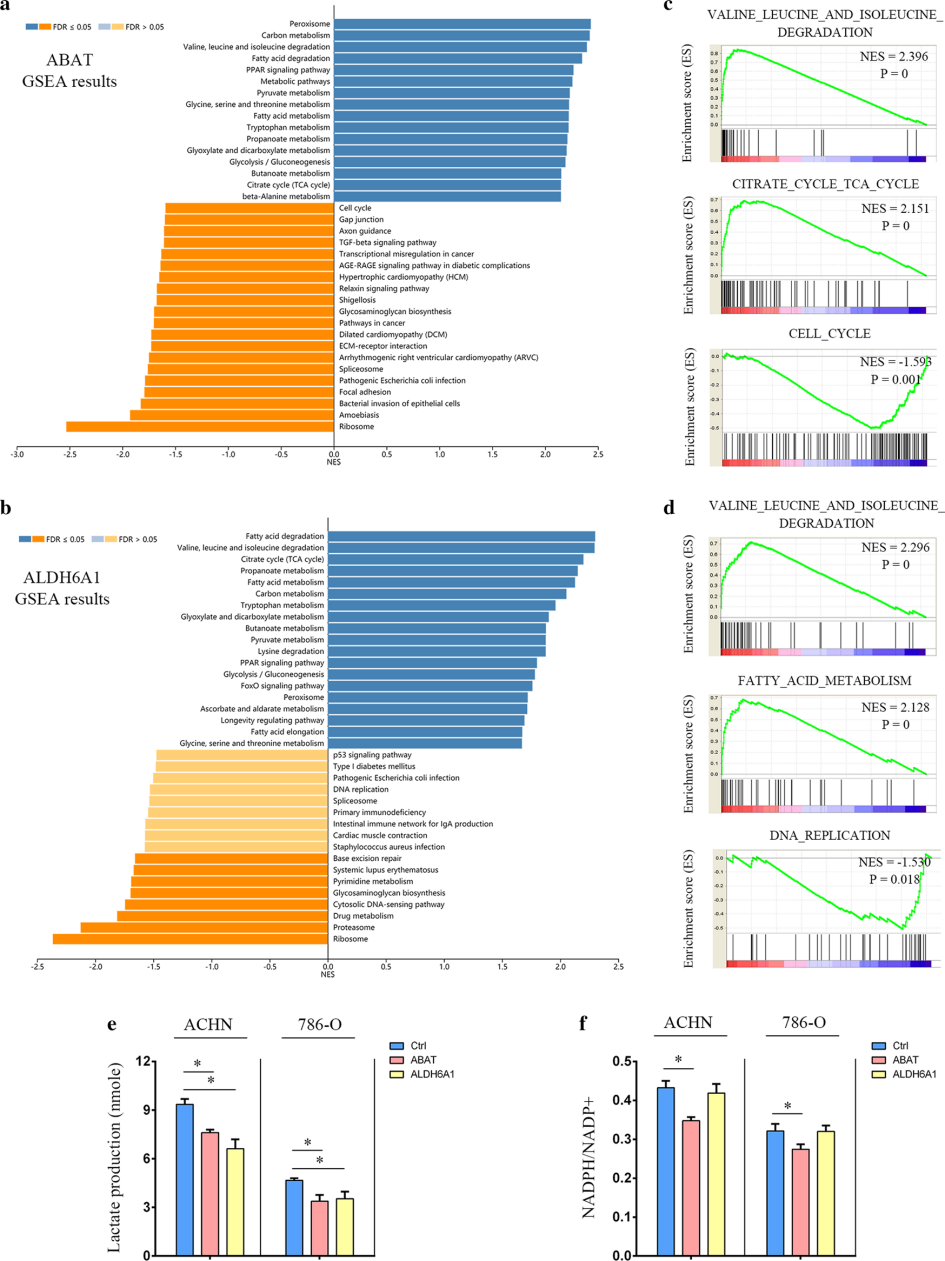
Effects of overexpression of ABAT and ALDH6A1 on ccRCC cell oncologic-metabolism. **a** LinkedOmics GSEA KEGG analysis of ABAT co-expression genes in TCGA-KIRC samples. **b** LinkedOmics GSEA KEGG analysis of ALDH6A1 co-expression genes in TCGA-KIRC samples. **c** Three KEGG pathways of ABAT. **d** Three KEGG pathways of ALDH6A1. **e** The lactate levels were measured. **f** The NADPH/NADP + ratios were measured. All the experiments were repeated three times; *p*-values were calculated compared with that of the control cells (Ctrl), and measured with Student’s t-tests; *p < 0.05

To investigate the effects of metabolic enzymes ABAT and ALDH6A1 on ccRCC metabolism and the role of gene abnormalities in driving ccRCC tumorigenesis, TCGA data were used to analyze gene set enrichment analysis (GSEA) of ABAT and ALDH6A1. Besides the valine, leucine, and isoleucine degradation pathway, the citrate cycle (TCA cycle) and fatty acid degradation correlated with downregulated levels of ABAT and ALDH6A1 (Fig. 6a, b). These metabolism pathways are known to be dysregulated in cancers, especially in ccRCC. Cell-proliferation-related pathways were found to be negatively correlated with ABAT and ALDH6A1, such as the cell cycle pathway with ABAT (Fig. 6c) and DNA replication pathway with ALDH6A1 (Fig. 6d). Increased production of lactic acid and NADPH are prominent characteristics of the metabolic remodeling of tumor cells [25]. To further confirm the link between ABAT and ALDH61 expression and metabolic modeling in ccRCC, we measured lactate levels and the NADPH/NADP + rate in ACHN and 786-O cells. We found that upregulated ABAT or ALDH6A1 impaired lactate production (Fig. 6e), and ABAT overexpression decreased the ratio of NADPH/NADP + (Fig. 6f). These data suggest that overexpression of ABAT or ALDH6A1 impairs cell oncologic-metabolism.

### Transcription factor HNF4A regulates ABAT and ALDH6A1 gene expression

**Fig. 7:**
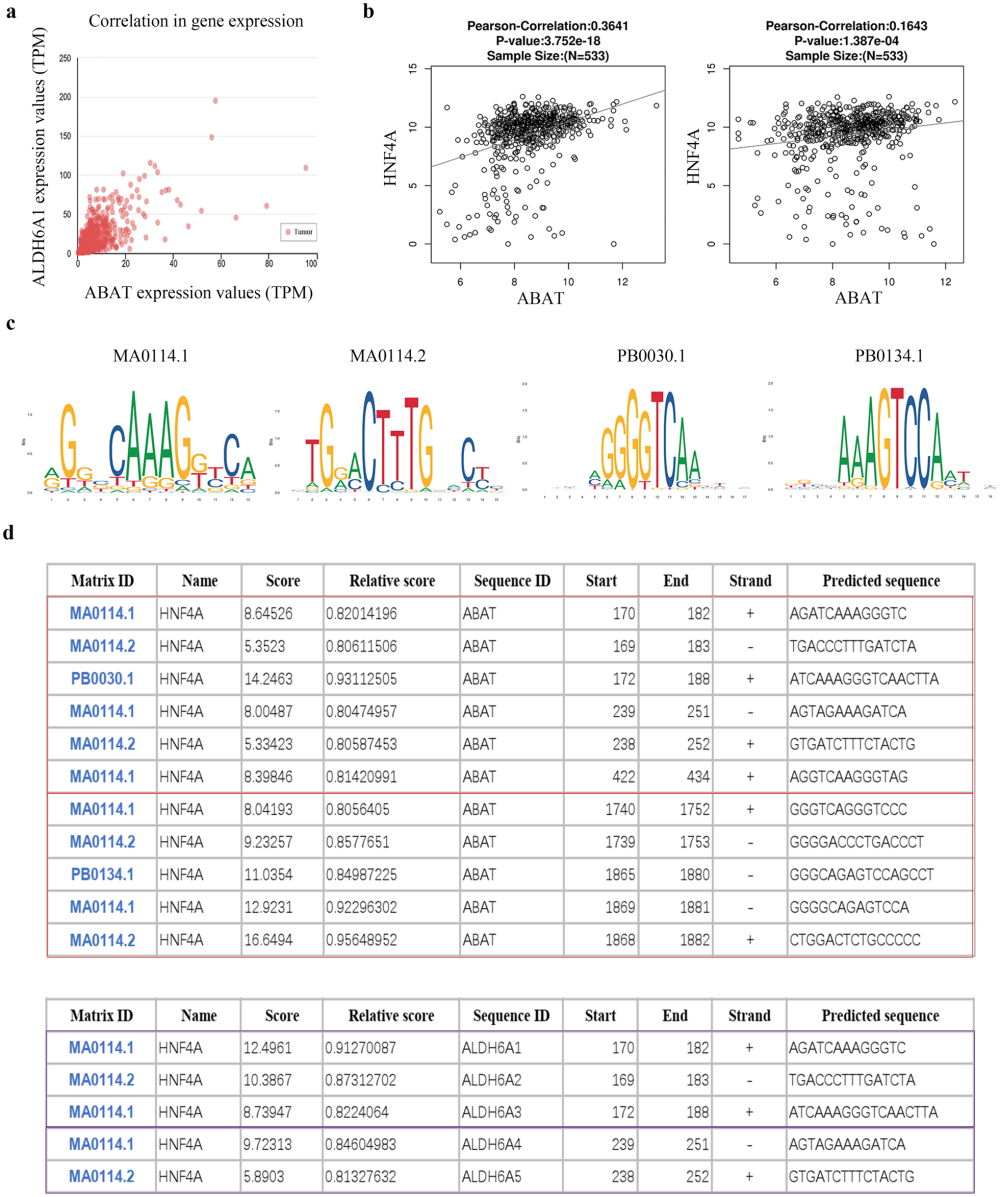
Identification candidate transcription factor related to the differential ABAT and ALDH6A1 expression. **a** UALCAN revealed significant positive correlation between ABAT and ALDH6A1 in TCGA-KIRC samples. **b** LinkedOmics revealed positive correlation between HNF4A and ABAT or ALDH6A1 in TCGA-KIRC samples. Pearson correlation coefficient and corresponding *p*-values are shown. **c** Detailed information of HNF4A binding matrix. **d** Binding sites of HNF4A on ABAT and ALDH6A1 promoter

In UALCAN, ALDH6A1 is the most expression-related gene of ABAT through Pearson correlation analysis, with a Pearson correlation coefficient (Pearson-CC) = 0.66 (Fig. 7a). By analyzing the transcription factors binding to the ABAT and ALDH6A1 promoters, we found that transcription factor HNF4A could simultaneously regulate ABAT and ALDH6A1, and HNF4A has a positive correlation with ABAT and ALDH6A1 expression (Fig. 7b). HNF4A is a well-known suppressor of ccRCC [26]. The expression of HNF4A is significantly downregulated in ccRCC and loss of HNF4A promotes tumorigenesis in the kidney [27]. We analyzed the promoter sequence of 2000 bp before the transcription initiation site on Jaspar. Four common binding sequences of HNF4A were selected (Fig. 7c). In each of the promoter regions of ABAT and ALDH6A1, there are two regions with more transcription factor binding sites (Fig. 7d).

**Fig. 8:**
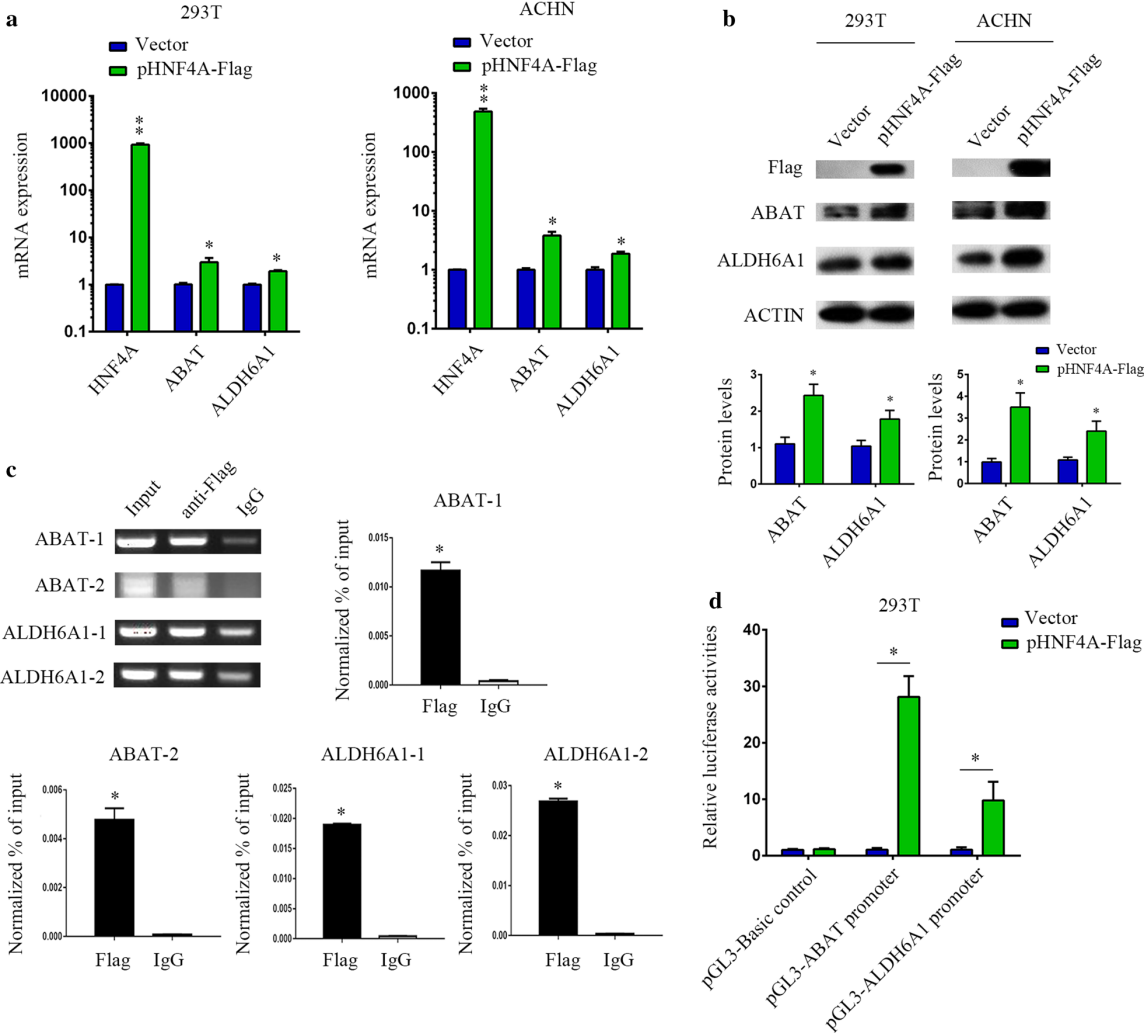
Functional effects of HNF4A on ABAT and ALDH6A1 expression. **a** HEK293T and ACHN cells were transiently transfected with pHNF4A-Flag or a control vector for 24 h. The mRNA levels of HNF4A, ABAT and ALDH6A1 were detected by qRT-PCR. The experiments were repeated three times. The *p*-value was measured using Student’s *t*-test; *p < 0.05, compared with the control cells. **b** HEK293T and ACHN cells were transiently transfected with pHNF4A-Flag or a control vector for 24 h. The protein levels of HNF4A-Flag, ABAT, and ALDH6A1 were detected by Western blotting. The experiments were repeated three times. **c** ChIP assay was used to analyze HNF4A binding to the two regions of ABAT and ALDH6A1 promoter in HEK293T cells. The *p*-value was measured using Student’s *t*-test; *p < 0.05, compared with IgG control. The experiments were repeated three times. **d** HEK293T cells were transiently transfected with pRL-TK and pGL3-Basic-control, pGL3-ABAT-promoter or pGL3-ALDH6A1-promoter and together with the empty vector or HNF4A expression vector for 24 h. Cell lysates were analyzed for luciferase activity. The experiments were repeated three times. The *p*-value was measured using Student’s *t*-test; *p < 0.05, compared with that of the empty-vector-transfected control cells

To examined whether ABAT and ALDH6A1 expressions in renal cell lines are regulated by exogenous HNF4A, HEK293T and ACHN cells were transfected with HNF4A expression vector or control plasmid. 786-O cells were not used in the transfection experiments because of the inability to achieve high transfection efficiency in 786-O cells. As shown in the Fig. 8a, exogenous overexpression of HNF4A induced the mRNA levels of endogenous ABAT and ALDH6A1 in HEK293T and ACHN cells. ABAT and ALDH6A1 protein levels were also significantly increased in HNF4A overexpressing cells (Fig. 8b). To further verify that HNF4A directly targets the promoter of ABAT and ALDH6A1, we performed a ChIP assay to assess the HNF4A-DNA interaction. Figure 8c demonstrates that Flag antibody co-immunoprecipitated the DNA sequence from the ABAT and ALDH6A1 promoter region by ordinary PCR. Real-time quantitative PCR showed similar results. Luciferase-based assays were subsequently used to verify the regulated role of HNF4A. As expected, in co-transfection experiments with pGL3-ABAT-promoter or pGL3-ALDH6A1-promoter and pHNF4A-Flag in HEK293T cells, we found that HNF4A increased the promoter activities of ABAT and ALDH6A1 (Fig. 8d). Together, these data indicate that HNF4A directly regulate expression of ABAT and ALDH6A1.

## Discussion

In this study, we first analyzed four of the GEO datasets and TCGA-KIRC data and found that the expression of ABAT and ALDH6A1 was decreased in ccRCC. The patients with low ABAT or ALDH6A1 gene expression had a worse survival period. Two ccRCC cell lines, ACHN and 786-O, were infected with ABAT or ALDH6A1 overexpressing lentivirus. The ability of cell clone formation was decreased in ABAT or ALDH6A1 overexpression cells, and the percentage of cell death was higher than in the control group. Transwell chamber experiments found that the cell migration ability of ABAT or ALDH6A1-overexpressed cells was inhibited. In order to understand the mechanism of overexpressing ABAT or ALDH6A1 in renal cancer cells, database analysis shows that ABAT and ALDH6A1 are mainly involved in oncologic metabolism. By measuring the lactic acid production and NADP/NADPH ratio of renal cancer cells, the results showed that after ABAT or ALDH6A1 overexpression, oncologic metabolism was impaired. We also demonstrated that ABAT and ALDH6A1 are directly regulated by a well-known tumor suppressor, transcription factor HNF4A.

In the present study, low ABAT expression is significantly associated with basal-like breast cancer aggressiveness by activating gamma-aminobutyric acid (GABA) signaling. Patients with ABAT deficiency display poor chemotherapy treatment outcome [28]. ABAT and ALDH6A1 are proved to be hub genes in association with metastasis risk and prognosis in hepatocellular carcinoma (HCC) [29]. Both of them are down-regulated in HCC [30, 31]. Our study identified that loss of ABAT and ALDH6A1 contributes to ccRCC tumor growth. Although the specific mechanisms of ABAT and ALDH6A1 in glutamate metabolism, glutathione GSH/GSSG determination, glucose uptake, and oxygen consumption should be determined in future research, our findings suggest that ccRCC patients might benefit from their influence on amino acid metabolism.

HNF4A inhibits proliferation in the stomach [32], colon [33] and kidney [27]. High expression of HNF4A leads to decreased growth of cancer cells. HNF4A-mediated inhibition of cell proliferation involves multifold mechanisms, including miRNA regulation and epigenetic repression of oncogenes, as shown using tissue-specific conditional knockout mice and knock-in studies, and significant cross talk with other cell cycle regulators including c-Myc and cyclin D1 [34]. The results of this study show that ABAT and ALDH6A1 are directly regulated by HNF4A, indicating that HNF4A suppresses ccRCC progression, at least in part, via the regulation of metabolic enzyme gene expression. Of cause, ABAT and ALDH6A1 may also be regulated by any microRNAs. In a future study, the expression levels of the related microRNAs need to be detected.

## Conclusion

ABAT and ALDH6A1 expression is significantly down-regulated in ccRCC tissues. Overexpression of ABAT or ALDH6A1 reduced cell proliferation and migration and impaired oncologic metabolism of renal cancer cells. Additionally, transcriptional factor HNF4A could be a potential mechanism affecting ABAT and ALDH6A1 expression.

## Supplementary information

**Additional file 1.** Additional tables.

## Data Availability

The datasets supporting the conclusions of this article are available in the Xena browser (https://xenabrowser.net/) repository.

## Abbreviations

ccRCC: Clear cell renal cell carcinoma
ChIP: Chromatin immunoprecipitation
DEGs: Differentially expressed genes
DMEM: Dulbecco’s modified Eagles medium
GEO: Gene Expression Omnibus
GSEA: Gene set enrichment analysis
HRP: Horseradish peroxidase
KEGG: Kyoto Encyclopedia of Genes and Genomes
mTOR: Mammalian target of rapamycin
NADPH: Nicotinamide adenine dinucleotide phosphate
RT-QPCR: Reverse transcription-quantitative polymerase chain reaction
TCGA: The Cancer Genome Atlas
TKI: Tyrosine kinase inhibitors
WST: Water-soluble tetrazolium salt
