# Supplementary material for: ABAT and ALDH6A1, regulated by transcription factor HNF4A, suppress tumorigenic capability in clear cell renal cell carcinoma
Source: J Transl Med. 2020 Feb 24;18:101. doi: 10.1186/s12967-020-02268-1 (PMC7038561; doi:10.1186/s12967-020-02268-1)
Supplement: Supplementary file 1 — Additional file 1. Additional tables. [file 12967_2020_2268_MOESM1_ESM.docx]

**Table S1.** **List of 50 clear cell renal cell carcinoma tissues**

| **Characteristics** | **N (%)** |
| --- | --- |
| **Gender** |  |
| Male | 35 (70%) |
| Female | 15 (30%) |
| **Age at surgery** |  |
| <60 | 30 (60%) |
| ≥60 | 20 (40%) |
| **Tumor extent** ^a^ |  |
| T1 | 41 (82%) |
| T2 | 7 (14%) |
| T3 | 2 (4%) |
| T4 | 0 (0%) |
| **Lymph node metastasis** ^a^ |  |
| N0 | 48 (96%) |
| ≥N1 | 2 (4%) |
| **Distant metastasis** ^a^ |  |
| M0 | 48 (96%) |
| M1 | 2 (4%) |
| **Tumor max diameter (cm)**^a^ |  |
| <7 | 41 (82%) |
| ≥7 | 9 (18%) |
| **Fuhrman grade**^b^ |  |
| G1 | 16 (32%) |
| G2 | 33 (66%) |
| G3 | 1 (2%) |
| G4 | 0 |

^a^According to 2009 Tumor‑Node‑Metastasis classifcation (7th) of malignant tumors by the International Union Against cancer.

^b^Based on the Fuhrman tumor grade system.

**Table S2.** **List of 29 clear cell renal cell carcinoma tissues**

| **Characteristics** | **N (%)** |
| --- | --- |
| **Gender** |  |
| Male | 15 (52%) |
| Female | 14 (48%) |
| **Age at surgery** |  |
| <60 | 15 (52%) |
| ≥60 | 14 (48%) |
| **Tumor extent** ^a^ |  |
| T1 | 22 (76%) |
| T2 | 4 (14%) |
| T3 | 2 (7%) |
| T4 | 1 (3%) |
| **Lymph node metastasis** ^a^ |  |
| N0 | 27 (93%) |
| ≥N1 | 2 (7%) |
| **Distant metastasis** ^a^ |  |
| M0 | 27 (93%) |
| M1 | 2 (7%) |
| **Tumor max diameter (cm)** ^a^ |  |
| <7 | 22 (76%) |
| ≥7 | 7 (24%) |
| **Fuhrman grade** ^b^ |  |
| G1 | 3 (10%) |
| G2 | 21 (73%) |
| G3 | 5 (17%) |
| G4 | 0 |

^a^According to 2009 Tumor‑Node‑Metastasis classifcation (7th) of malignant tumors by the International Union Against cancer.

^b^Based on the Fuhrman tumor grade system.
